# Multiple forms of balancing selection maintain inversion polymorphism

**DOI:** 10.1038/s41437-025-00780-y

**Published:** 2025-07-17

**Authors:** Margot Paris, Esra Durmaz Mitchell, Envel Kerdaffrec, Doriane Rubin, Cécile Spichtig, Felicitas Zurbriggen, Joël Becker, Hannah Augustijnen, Harshavardhan Thyagarajan, Eliane Zinn, Fanny Gagliardi, Elliot Gobet, Tristan Rey, Yvan Rime, Sofia Ribeiro Machado, Jeremias Bachmann, Noemi Sgammeglia, Paul Schmidt, Thomas Flatt

**Affiliations:** 1 Department of Biology, University of Fribourg, Fribourg, Switzerland; 2 Department of Biochemistry and Molecular Biology, University of Southern Denmark, Odense, Denmark; 3 Department of Biology, University of Pennsylvania, Philadelphia, PA USA

**Keywords:** Evolution, Genetics

## Abstract

Despite many examples of balanced inversion polymorphisms, little is known about how they affect fitness-related traits. This knowledge gap hampers our understanding of how they are selectively maintained as protected polymorphisms. Here, we study the effects of a cosmopolitan balanced inversion polymorphism in *D. melanogaster*, *In(3R)Payne*, on fitness components, including traits related to development, growth, reproduction, stress resistance, and adult survival. We find that the non-inverted standard (STD) chromosomal arrangement and the inverted (INV) arrangement behave like Mendelian alleles of a supergene, which affect a suite of complex fitness-related phenotypes. While the STD arrangement tends to have positive, mostly dominant effects on size-related traits, fecundity, fertility, stress resistance, and lifespan, the INV arrangement exhibits mostly recessive effects that are indicative of fitness costs. Yet, in favor of the balanced polymorphism, we observe overdominance for egg hatchability, egg-to-adult survival, pupal survival at 18 °C, developmental time, and male desiccation resistance. The most parsimonious explanation for these heterotic effects is that they are due to some form of multi-locus heterokaryotype advantage. We also find several instances of trait-, sex-, and temperature-dependent changes in the degree of dominance, suggesting a possible role of antagonistic selection with context-specific dominance reversals in maintaining the polymorphism. Moreover, genotype-by-environment interactions and parental effects appear to contribute as well. Together, our results suggest that multiple phenotypic modes of balancing selection are involved in maintaining the inversion polymorphism.

## Introduction

In 1946, Sewall Wright and Theodosius Dobzhansky provided the first unambiguous experimental demonstration that some naturally occurring chromosomal inversion polymorphisms of *Drosophila pseudoobscura* affect Darwinian fitness and that they can be stably maintained in population cages under constant conditions by some form of balancing selection (Wright and Dobzhansky 1946). Since their pioneering study, numerous examples of adaptive inversion polymorphisms have been documented in a wide variety of organisms (e.g., reviewed in Krimbas and Powell 1992; Lewontin et al. 2003; Hoffmann et al. 2004; Hoffmann and Rieseberg 2008; Kirkpatrick 2010; Wellenreuther and Bernatchez 2018; Faria et al. 2019; Fuller et al. 2019; Kapun and Flatt 2019; Huang and Rieseberg 2020; Berdan et al. 2023).

Despite this large body of work, however, phenotypic effects are either poorly understood or unknown for the great majority of inversions. In particular, our understanding of how inversions affect fitness components remains limited, with some notable exceptions, especially in the genus *Drosophila* (reviewed in Sperlich et al. (1986); Krimbas and Powell 1992; Kapun and Flatt 2019), but also, for example, in the seaweed fly *Coelopa frigida* (Butlin, Day (1984); Mérot et al. 2020), stick insects of the genus *Timema* (Nosil et al. 2023), the marine snail *Littorina saxatilis* (Koch et al. 2021), the ruff *Philomachus pugnax* (Küpper et al. 2016), the zebra finch *Taeniopygia guttata* (Knief et al. 2016, 2017; Pei et al. 2023), and the monkey flower *Mimulus guttatus* (Lowry and Willis 2010) (e.g., see Wellenreuther and Bernatchez 2018 for a taxonomically broader overview). This gap in our knowledge impedes our understanding of how selection acts on inversions and, notably, how balancing selection maintains polymorphic inversions in natural populations (Berdan et al. 2023). This is further complicated by the fact that there exist numerous distinct forms of balancing selection (e.g., see Ruzicka et al. 2025).

Here, we investigate how a cosmopolitan inversion polymorphism in *Drosophila melanogaster*, *In(3* *R)Payne* (or, in short, *In(3* *R)P or 3RP*), known to be maintained by balancing selection (e.g., see Kapun and Flatt 2019; Kapun et al. 2023, and references therein), affects fitness-related traits. This ~8-Mb large paracentric inversion spans ∼1200 genes on the right arm of the third chromosome (*3* *R*) and comprises two alternative chromosomal arrangements, the ancestral non-inverted (standard = STD) and the derived inverted (INV) arrangement. This polymorphism is relatively common globally, with the INV arrangement occurring at intermediate frequencies in low-latitude populations while being absent at high latitudes (e.g., Mettler et al. 1977; Knibb et al. 1981; Knibb 1982; Lemeunier and Aulard 1992; Matzkin et al. 2005; Anderson et al. 2005; Kapun et al. 2016a; Kapun and Flatt 2019; Kapun et al. 2023).

Previous work has provided evidence that *3RP* is subject to spatially varying selection along latitudinal clines, temporally (seasonally) varying selection, as well as negative frequency-dependent selection (e.g., Nassar et al. 1973; Sanchez-Refusta et al. 1990 Lemeunier and Aulard 1992; Fabian et al. 2012; Kapun et al. 2016a; Kapun and Flatt 2019; Kapun et al. 2020; Machado et al. 2021; Kapun et al. 2023). However, it remains largely unclear how these different forms of balancing selection maintain this polymorphism and whether and how they interact.

Several prior studies have examined phenotypic effects of *3RP*, either by using associations between genetic markers within the inversion and traits or by assaying chromosome extraction lines, finding effects on size-related traits, pigmentation, starvation resistance, heat resistance, cold susceptibility, and lifespan (Weeks et al. 2002; Anderson et al. 2003; Rako et al. 2006; Kennington et al. 2007; Takahashi and Takano-Shimizu 2011; Kapun et al. 2016b; Durmaz et al. 2018). Importantly, however, none of these studies specifically investigated the phenotypic effects of the inversion heterokaryotype (but cf. Barnes 1983). As inversions strongly reduce the effective rate of crossing over between the two arrangements in the heterokaryotypic state, and as reduced recombination between adaptive loci on the inverted haplotype can confer a selective advantage to the heterokaryotype, for example when the loci involved are subject to overdominance (e.g., Dobzhansky 1949, 1950, 1951, 1970; Charlesworth and Charlesworth 1973; Charlesworth 1974; Kirkpatrick and Barton 2006; Durmaz et al. 2020; Charlesworth and Flatt 2021; Berdan et al. 2023; Charlesworth 2024), assessing how inversion heterokaryotypes affect fitness-related traits is particularly important. Yet, even though inversions in this system have been studied extensively (reviewed in Lemeunier and Aulard 1992; Kapun and Flatt 2019), only a few studies to date have reported heterokaryotype superiority in *D. melanogaster* (e.g., Watanabe and Watanabe 1973; Kamping and van Delden 1999).

Here, we assay *3RP* homo- and heterokaryotypes for 12 phenotypes associated with development, size, reproduction, stress resistance, and lifespan, and observe that this inversion behaves like a ‘coadapted gene complex’ (e.g., Dobzhansky 1949, 1950, 1951, 1970; Charlesworth 1974; Charlesworth and Flatt 2021) or a ‘supergene’ (e.g., Thompson and Jiggins 2014; Schwander et al. 2014; Berdan et al. 2022) with multifarious effects on a large suite of fitness components.

Notably, our results suggest that this chromosomal inversion polymorphism might be subject to several forms of balancing selection, including overdominant selection (e.g., Ruzicka et al. 2025), antagonistic selection with context-dependent reversals of dominance (e.g., Grieshop et al. 2024), genotype-by-environment interactions (Felsenstein 1976; Gillespie and Turelli 1989), as well as parental ‘storage’ effects (Yamamichi and Hoso 2017).

## Materials and Methods

### Fly stocks and maintenance

Isofemale lines originated from three populations in Florida, USA (Homestead, Jacksonville, and Miami; cf. Kapun et al. 2016b; DiVito Evans et al. 2023) and were maintained as stocks at 18 °C, 60% relative air humidity (RH), and 12 h:12 h [h] light:dark (L:D) photoperiod. Before chromosome extraction, isofemale lines were screened for the presence of 6 polymorphic inversions (*In(2* *L)t*, *In(2* *R)NS*, *In(3* *L)P*, *In(3* *R)P*, *In(3* *R)K*, *In(3* *R)Mo*), using PCR primers and protocols from Matzkin et al. (2005) and Corbett-Detig and Hartl 2012. Lines that either carried none of the six inversions tested or that carried only *In(3R)P* were selected for isolation of *3RP* STD and INV chromosomes, respectively. Chromosomal isolation was performed following the approach of Kapun et al. (2016b), using a compound balancer stock for the second and third chromosome (*SMB6*; *TM6B*) in an *ebony* (*e*^1^) mutant background. Briefly, males from the isofemale line were individually crossed to balancer stock females. Offspring of each cross carrying the balancer (and hence carrying a wild-type third chromosome of interest over the balancer) were selected at the pupal stage based on the “tubby” phenotype associated with the dominant *Tubby* (*Tb*) marker mutation carried by *TM6B*. Selected offspring were backcrossed to the balancer to amplify each isolated chromosome, and diagnostic PCRs were used to determine the *3RP* karyotype. In total, we isolated 25 STD and 25 INV chromosomes (15 and 18 from Homestead, 9 and 4 from Jacksonville, and 1 and 2 from Miami for STD and INV, respectively). The 50 isolated chromosomes were maintained as stocks over the compound balancer under standard laboratory conditions at 25 °C, 60% RH, and 12 h:12 h L:D.

To investigate the phenotypic effects of the inversion, we established large, triplicate population cages as panmictic and recombining but chromosomally monomorphic (homokaryotypic) populations at a large population size (~10,000–12,000 flies per cage) under standard lab conditions (see above). Each of these monomorphic population cages was initiated by mixing the 25 chromosomal lines of a given karyotype (either STD or INV) in equal proportions; in the F1, we selected against all individuals carrying the dominant *Tb* marker (and hence the compound balancer) at the pupal stage, thus only retaining wild-type flies for further breeding. In these recombining monomorphic populations, the 2nd, 3rd and *Y* chromosomes come from the Florida wild-type background; the *X* and 4th dot chromosomes are a mix of the Florida and balancer backgrounds; and the maternally transmitted mitochondrial genome comes from the balancer background. This design allowed us to generate outbred populations of the *3RP* homokaryotypes (STD/STD, INV/INV) and, at the same time, to efficiently create *3RP* heterokaryotypic (STD/INV and INV/STD) flies by using (reciprocal) mass crosses between flies from STD vs. INV cages. The first phenotypic assays were carried out after 15 generations of monomorphic cage culture (see Table S1 for details).

### General phenotyping methods

We assayed 12 fitness components (reviewed in Flatt 2020) related to (i) development (egg hatchability, egg-to-adult survival [viability], pupal survival [pupa-to-adult survival], and the time course of egg-to-adult emergence [to assess development rate and time], measured without distinguishing the sexes); (ii) adult female and male size (dry weight, wing area, and femur length); (iii) female reproduction (fertility [number of viable offspring] over 30 days [d] of adulthood, and daily per-capita fecundity at 20–22 d of adulthood); and (iv) stress and survival traits (survival upon starvation [starvation resistance], survival upon desiccation [desiccation resistance], and lifespan), all assayed in both females and males.

Assays were performed on mated flies using plastic vials or bottles containing standard (cornmeal/sugar/yeast/agar) food medium (8 mL and 25 mL for vials and bottles, respectively) maintained at constant temperature (25 °C, which represents the standard assay temperature, and, depending on the trait also at 18 °C), 60% RH and 12 h:12 h L:D; survival assays were performed in demography cages (see below), with food vials attached, using the same conditions. The positions of vials, bottles, and demography cages were randomized to avoid confounding thermal position effects inside incubators or climate chambers.

Traits were measured on *3RP* karyotypes produced from mass crosses using the aforementioned panmictic population cages: the STD/STD homokaryotype, the INV/INV homokaryotype, and two heterokaryotypes from reciprocal crosses, namely INV/STD (INV dam [mother], STD sire [father]) and STD/INV flies (STD dam [mother], INV sire [father]). Flies from INV and STD cages were reared in bottles under controlled low-density conditions, with ~10 eggs/mL of medium, before sexing at the pupal stage. For each cross, 75 females and 75 males were allowed to mate and lay eggs on Petri plates with standard medium in replicated 1 L oviposition chambers maintained at 25 °C or 18 °C; F1 offspring from these crosses were phenotyped at the same temperatures. For assays of developmental and size traits, eggs were collected from Petri plates of oviposition chambers and placed into vials at controlled low density (30 eggs per vial; ~3.8 eggs/mL medium), with the help of a Zeiss Stemi DV4 stereo microscope. The same procedure was used for assays of reproductive, stress, and survival traits, except that eggs were transferred to bottles at a density of ~10 eggs/mL of medium.

Details on assay generations and the numbers of replicates and flies assayed for each karyotype and trait are provided in Table S1, and the phenotypic raw data in Table S2.

### Developmental traits

Egg hatchability was measured at both 25 °C and 18 °C as the percentage of eggs that hatched; batches of 5 females and 5 males from INV and STD cages were used as parents to produce eggs of each karyotype (i.e., 5 INVx 5 INV; 5 INVx 5 STD; 5 STDx 5 INV; 5 STDx 5 STD), and eggs were reared in bottles under controlled density conditions. Adults were allowed to oviposit in fresh vials for 15 h and 17 h at 25 °C and 18 °C, respectively. Using a Zeiss Stemi DV4 stereo microscope, we counted the total number of eggs per vial before egg hatching and then assessed the number of unhatched eggs 48 h after oviposition. For assays of egg-to-adult survival (viability) and, separately, of pupal survival at 25 °C and 18 °C, we collected batches of 30 eggs from oviposition chambers and placed them into individual vials. Viability was estimated per replicate vial as the percentage of adults emerging from the initial 30 eggs. Similarly, pupal survival was calculated per vial as the proportion of pupae that eclosed as adults from the initial 30 eggs. Development rate and time were quantified by analyzing the proportion of adult emergence (eclosion) over time (see below). Batches of 30 eggs were placed in replicate vials and allowed to develop until adult emergence at 25 °C. Individual eclosed adults were collected and sexed at 21 time points, from 198 to 333 h after egg laying, with a minimum of 2 h between 13 time points around the peak of eclosion (at 198, 213, 216, 218, 220, 222, 224, 226, 228, 230, 232, 234, 236, 238, 240, 242, 246, 261, 285, 309, and 333 h).

### Size-related traits

For assays of size-related traits, we reared flies of all four karyotypes at a controlled low density of 30 eggs per vial. Adult flies were collected daily and kept in new vials for 5 d, allowing them to sexually mature and reach their adult size before storing them at −20 °C prior to measurements. To measure dry weight, flies were sorted by sex after 24 h at −20 °C, dried in groups of five in an oven at 90 °C for 72 h, and weighed using a Mettler Toledo AG204 microbalance. For measurements of wing area and femur length, the left wing and the first right leg of flies were removed and mounted on microscope slides using CC/Mount tissue mounting medium from Sigma-Aldrich. Images of legs and wings were captured using a Leica DFC425C digital camera connected to a Leica MZ6 microscope and analyzed using ImageJ v1.53t software. For wings, we measured the area between 5 landmarks, and for femurs, the distance between 2 landmarks (see Supporting Information, Fig. S1). All morphometric measurements were repeated twice and averaged before statistical analysis.

### Reproductive traits

We performed two assays of female reproductive output: one focusing on fertility over time, and the other on age-specific fecundity at a specific, relatively late age of adulthood. First, we measured daily per-capita female fertility, the number of viable adult offspring produced per female per day, at 25 °C over the first 30 d of adulthood, by following individual females over time. In addition to daily fertility estimates, we also estimated total per-capita fecundity over the entire 30-d period from the time series. Flies were collected and sexed at the pupal stage after having been reared in bottles under controlled low-density conditions. One female and two males of the same karyotype were placed in replicate vials for mating and oviposition. Flies were transferred to new vials every day until day 6 and then every two days until day 30. Second, we estimated daily per-capita fecundity (i.e., the number of eggs per female) at a relatively late age (20–22 d) of adulthood. We chose this age to validate the results of our previous fertility time-course analyses (see above) which had suggested karyotypic differences in fertility at relatively late ages (see Fig. 3B). We carried out these late-age daily fecundity assays at 25 °C and 18 °C using batches of 5 females and 5 males of the same karyotype. At 20–21 d of adulthood, flies were transferred to fresh vials and allowed to oviposit for 15 h at 25 °C and for 17 h at 18 °C. Eggs were counted under a Zeiss Stemi DV4 stereo microscope before hatching and larval development. From these egg count data, we calculated daily per-capita fecundity for the adult age range between 20 and 22 days.

### Stress and survival traits

For assays of starvation resistance at 25 °C, 4–5 day-old adults of both sexes were transferred to 1 L demography cages (see Tatar et al. 2001), without food, in batches of ~300 flies using a calibrated pipette tip. In each cage, flies had access to a dense cellulose acetate flug (25 mm diameter) soaked with water and changed every 2 day to avoid desiccation. Dead flies were removed, sexed, and counted every 4 h between 20 and 56 h of starvation at the peak of mortality, then every 12 h between 56 and 92 h, and finally every 24 h until all flies had died after 524 h of starvation. For assays of desiccation resistance, 4–5 day-old adults of both sexes were transferred to cages in batches of ~275 flies using a calibrated tip and maintained at 25 °C with 40% RH without food and water (note that such assays inevitably impose both desiccation and starvation). Dead flies were removed, sexed, and counted at 10, 12, 14, 16, 18, 20, 23, 25, 27, and 29 h after the start of the assay. To measure adult lifespan, freshly eclosed adults were sexed under light CO_2_ anesthesia, and mixed-sex batches of ~100 adults were transferred to replicate cages maintained at 25 °C. The two heterokaryotypes (STD/INV, INV/STD) were mixed in equal proportions and not investigated separately. Dead flies were removed, sexed, and counted, and food vials attached to the cages were changed every Monday, Wednesday, and Friday until all flies had died in the cages. In all stress and survival assays, any escaped or accidentally killed flies were censored.

### Statistical analyses

Statistical analyses were performed with R v4.2.0 (R Core Team 2022). The R code used for the statistical models is given in Table S1. Box plots were made with the geom_boxplot option in ggplot2; in each plot, the lower line of the rectangle represents the 25th percentile, the upper line the 75th percentile, and the line inside the rectangle represents the median.

For all analyses shown in the main text, and to analyze the degree of dominance (see below), we pooled INV/STD and STD/INV heterokaryotypes (HET) from both cross directions; analyses of differences between cross directions, which are indicative of parental effects, are given in the Supplementary Information (Figs. S3, S4; also see Table S1).

To analyze the effects of *3RP* karyotype on hatchability, viability, pupal survival, weight, wing area, femur length, fertility, and fecundity, we used non-parametric Kruskal-Wallis tests, i.e., one-way analysis of variance (ANOVA) on rank-transformed data, followed by Dunn’s posthoc tests for pairwise comparisons and Benjamini-Hochberg corrections (Benjamini and Hochberg 1995) for multiple testing, using the R packages stats and FSA v0.9.5 (Ogle et al. 2023).

To analyze fertility trajectories over time, we used repeated-measures multivariate analysis of variance (MANOVA). Specifically, we employed modified ANOVA-type statistic MATS (Friedrich and Pauly 2018), with *p*-values based on a wild bootstrap approach with Rademacher weights, developed for repeated measures designs using the multRM function of the R package MANOVA.RM v0.5.4 (Friedrich et al. 2025). In this analysis, a significant karyotype-by-time interaction term indicates that the karyotypes differ in their fertility trajectories over time. Analyses were performed for the entire 30-day period as well as for 10-day intervals (days 1–10, 11–20, 21–30). For analyses of 10-day intervals, *p*-values of pairwise comparisons between karyotypes were corrected for multiple testing using the Benjamini-Hochberg method.

Results for time-to-event data (developmental rate or time, survival upon starvation, survival upon desiccation, and lifespan) were visualized using Kaplan–Meier survival curve estimates with the survfit() and ggvurvplot() functions of the survival v3.5-5 (Therneau 2023) and survminer v0.4.9.999 (Kassambara et al. 2021) packages. The effects of karyotype on development time, starvation resistance and desiccation resistance were analyzed using pairwise comparisons with log-rank tests and Benjamini-Hochberg corrections for multiple testing with the pairwise_survdiff() function of the survminer v0.4.9.999 package (Kassambara et al. 2021). To analyze adult lifespan, we used the Peto and Peto modification (Peto and Peto 1972) of the Gehan-Wilcoxon test (Gehan 1965) implemented in the pairwise_survdiff() function, followed by Benjamini-Hochberg corrections for multiple testing. This test is more appropriate than the log-rank test when hazards are not proportional over time (e.g., when survival curves cross) and weights early deaths more strongly.

### Estimation of dominance coefficient *h*

For each trait, we quantified the dominance coefficient *h* of the INV arrangement according to the formula *h* = (mean [STD/STD] - mean [HET]) / (mean [STD/STD] – mean [INV/INV]) (Bourguet et al. 2000).

We classified the degree of dominance *h* as follows (e.g., Falconer and Mackay 1996): *h* = 0 means complete recessivity; *h*-values between 0 and 0.5 indicate partial recessivity; *h* = 0.5 defines additivity; *h*-values between 0.5 and 1 represent partial dominance; and *h* = 1 indicates complete dominance. Values falling outside the range between 0 and 1 represent underdominance (*h* < 0; not observed in our study) or overdominance (*h* > 1). For the observed instances of overdominance (see Results section), we extended the ratio scale above 1 and estimated *h*-values by calculating the ratio (trait value of the heterokaryotype divided by the average trait value of the two homokaryotypes).

In all cases of overdominance, the trait value of the heterokaryotype was *significantly greater* than the trait values of *both* homokaryotypes, thus providing statistical evidence for overdominance. In addition, we estimated relative fitness-component effects by assigning a maximum value of 1 to the heterokaryotype and calculating the relative effects (<1) of the two homokaryotypes (see Charlesworth 2024 for similar estimates).

## Results

### The heterokaryotype exhibits overdominance for pre-adult fitness components

**Fig. 1:**
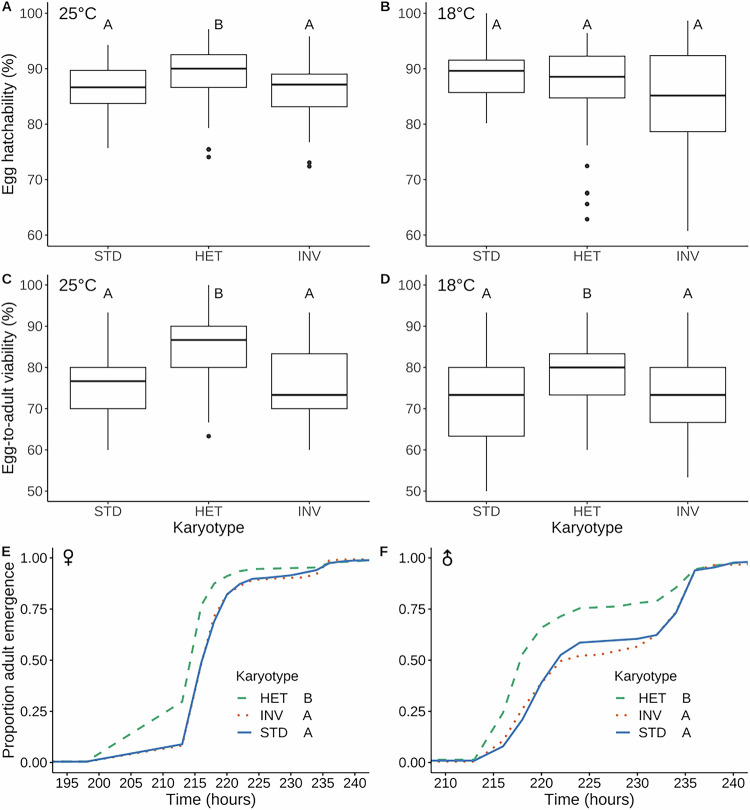
Effects of *3RP* on developmental traits. Percentage of egg hatchability at 25 °C (**A**) and 18 °C (**B**). Percentage of egg-to-adult survival at 25 °C (**C**) and 18 °C (**D**). Proportion of adult emergence over time for females (**E**) and males (**F**). Different letters indicate significant pairwise differences (*P* < 0.05) between karyotypes after multiple-testing correction.

We first examined how *3RP* affects pre-adult traits (Fig. 1; Tables 1, 2; Table S1). We observed significant heterokaryotype advantage (dominance coefficient *h* > 1; Tables 1, 2) for egg hatchability at 25 °C (Fig. 1A; without differences among karyotypes at 18 °C, see Fig. 1B), pupal survival at 18 °C (see Supplementary Information, Fig. S2; without differences among karyotypes at 25 °C), egg-to-adult survival at both 18 °C and 25 °C (Fig. 1C, D), and for both female and male developmental rate at 25 °C (Fig. 1E, F). Thus, in terms of these major determinants of pre-adult fitness, the *3RP* heterokaryotype seems to be superior to the STD/STD and INV/INV homokaryotypes, with a few of these effects (hatchability, pupal survival) depending on temperature, i.e., exhibiting genotype (karyotype) × environment interactions (GxE) (Fig. 1; Tables 1, S1; also see below). While the effect of heterokaryotype superiority was relatively small for hatchability and development rate (~1–3%), it was quite large for viability (6–11%, depending on assay temperature) (Table 2).

**Table 1:** Overview of the effects of *In(3R)P* on fitness components.

Phenotypic trait	Direction of effect	Degree of dominance (*h*) of INV	Putative fitness effect of INV or polymorphism	Dominance changes between sexes
Egg hatchability (25 °C)	HET > STD = INV	**overdominant** (*h* = 1.03)	**+**	NA
Viability (18 °C)	HET > STD = INV	**overdominant** (*h* = 1.12)	**+**	NA
Viability (25 °C)	HET > STD = INV	**overdominant** (*h* = 1.07)	**+**	NA
Developmental rate (F; 25 °C)	HET > STD = INV	**overdominant** (*h* = 1.01)	**+**	NA
Developmental rate (M; 25 °C)	HET > STD = INV	**overdominant** (*h* = 1.02)	**+**	NA
Pupal survival (18 °C)	HET > STD = INV	**overdominant** (*h* = 1.02)	**+**	NA
Dry weight (F)	STD > HET > INV	partially recessive (*h* = 0.31)	**-**	NA
Wing area (F)	STD > HET = INV	dominant for small size (*h* = 1)	**-**	NA
Wing area (M)	STD > HET > INV	partially dominant (*h* = 0.56)	**-**	NA
Femur length (F)	STD = HET > INV	recessive (*h* = 0)	**-**	sex-dependent dominance reversal
Femur length (M)	STD > HET = INV	dominant for small size (*h* = 1)	**-**	
Total fertility (over 30 days)	STD = HET; STD = INV; HET > INV	?	?	NA
Age-specific fertility (age 21–30 days)	STD = HET > INV	recessive (*h* = 0)	**-**	NA
Daily per-capita fecundity (age 20–22 days) (25 °C)	STD = HET > INV	recessive (*h* = 0)	**-**	NA
Daily per-capita fecundity (age 20–22 days) (18 °C)	STD = HET; STD > INV; HET = INV	partially recessive (*h* = 0.4)	**-**	NA
Starvation resistance (F)	STD > HET > INV	additive (*h* = 0.5)	**-**	NA
Starvation resistance (M)	STD = HET; STD > INV; HET = INV	?	**-**	NA
Desiccation survival (F)	STD = HET > INV	recessive (*h* = 0)	**-**	sex-dependent change of dominance
Desiccation survival (M)	HET > STD = INV	**overdominant** (*h* = 1.20)	**+**	
Lifespan (F)	STD = HET > INV	recessive (*h* = 0)	**-**	NA
Lifespan (M)	STD > INV; STD = HET; INV = HET	recessive (*h* = 0)	**-**	NA

For each trait the table gives the directionality of the phenotypic effect with regard to the three karyotypes, the degree of dominance with respect to the INV arrangement (with significant cases of overdominance highlighted in boldface; also see Table 2); the potential fitness effect of the INV arrangement or the polymorphism; and changes in the degree of dominance between temperatures or sexes.

Symbols: ‘+’ refers to putatively positive fitness effects; ‘-‘ denotes potentially negative effects.; ‘?’ means ‘not be determinable from the data’. For further details see the main text and Table S1. The results for hatchability at 18 °C (Fig. 1B), pupal survival at 25 °C (see Supplementary Information Fig. S1), and for male dry weight (see Table S1) are not given here as there were no significant differences among the karyotypes for these traits (see Table S1 for details). *F* female, *M* male, *INV* INV/INV inverted homokaryotype, *STD* STD/STD non-inverted, ‘standard’ homokaryotype, *HET* STD/INV and INV/STD heterokaryotype, *h* dominance coefficient, *NA* not applicable (i.e., does not apply, or could not be observed, e.g., because the trait was only measured in one sex or at a single temperature).

**Table 2:** Relative fitness-component effects of the *In(3R)P* karyotypes for fitness components showing statistically significant overdominance (also see Table 1 and main text for details).

Karyotype	Rel. egg hatchability (25 °C)	Rel. viability (18 °C)	Rel. viability (25 °C)	Rel. dev. rate (F; 25 °C)	Rel. dev. rate (M; 25 °C)	Rel. pupal survival (18 °C)	Rel. desiccation survival (M)
STD	0.97	0.93	0.89	0.99	0.98	0.98	0.83
HET	1	1	1	1	1	1	1
INV	0.97	0.94	0.89	0.99	0.97	0.99	0.83

(Relative effects were estimated by assigning a maximum value of 1 to the heterokaryotype and calculating the relative effects (<1) of the two homokaryotypes. While some homokaryotypic effects were small (∼1–3%), others were relatively large (6–7% for viability at 18 °C) or very large (11% for viability at 25 °C; 17% for male desiccation survival).

*STD* STD/STD non-inverted, ‘standard’ homokaryotype, *HET* STD/INV and INV/STD heterokaryotype, *INV* INV/INV inverted homokaryotype, *Rel.* relative, *dev.* developmental, *F* female, *M* male.

### The INV arrangement confers smaller body size

**Fig. 2:**
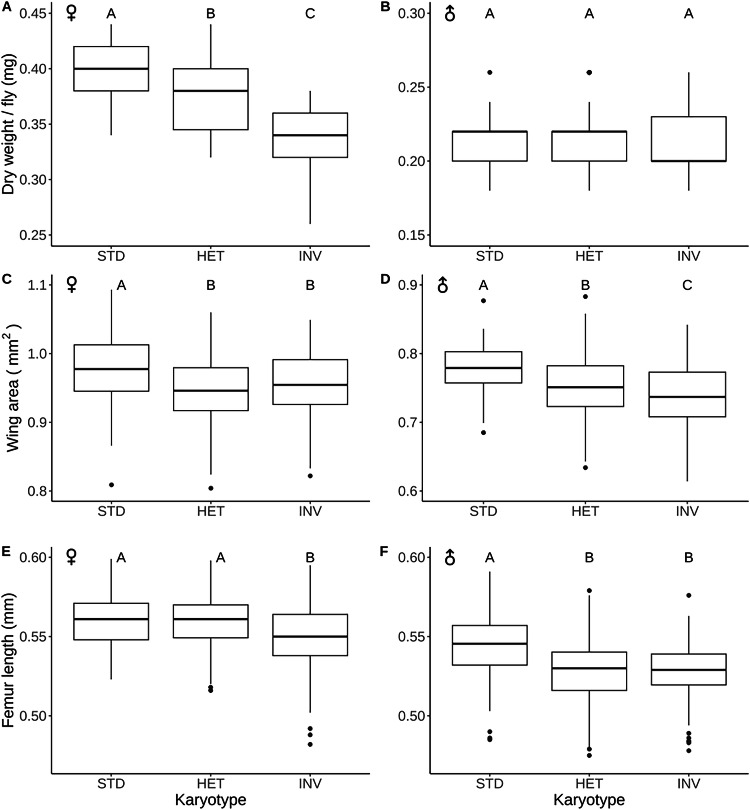
Effects of *3RP* on size-related traits. Dry weight for females (**A**) and males (**B**). Wing area for females (**C**) and males (**D**). Femur length for females (**E**) and males (**F**). Different letters indicate significant pairwise differences (*P* < 0.05) between karyotypes after multiple-testing correction.

We next measured several size-related traits, with body size representing a major fitness correlate in *D. melanogaster* (e.g., De Jong and Bochdanovits 2003; Flatt 2020). Generally, STD arrangement flies were larger than INV arrangement flies for all proxies of size (Fig. 2; Tables 1, S1). Dry weight differed significantly between karyotypes only for females, with rank order STD > HET > INV (Fig. 2A; Table 1). For wing area, INV was dominant over STD in terms of conferring smaller size in females and partially dominant in males (Fig. 2C, D; Table 1). For female femur length, INV had a recessive effect, while in males, INV was dominant in terms of conferring smaller size, thus indicating a sex-dependent reversal of dominance (Fig. 2E, F; Tables 1, S1).

### STD has a higher reproductive output than INV

**Fig. 3:**
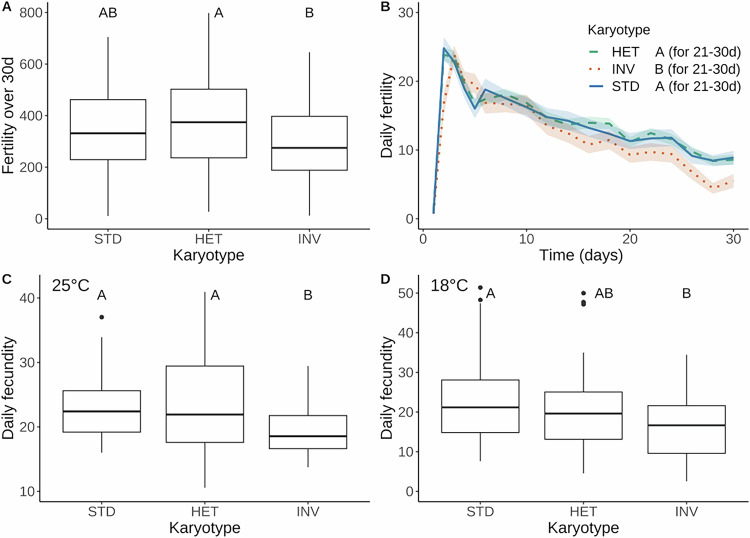
Effects of *3RP* on reproduction. Total per capita fertility (number of viable offspring per female) over 30 days of adulthood (**A**). Per-capita fertility over time; the colored envelopes around the curves represent standard errors of the mean (**B**). Daily per-capita fecundity at 20–22 days at 25 °C (**C**) and 18 °C (**D**). Different letters indicate significant differences (*P* < 0.05) between karyotypes after multiple-testing correction. For fertility over time (**B**), the letters indicate differences between karyotypes for the period 21–30 days.

HET females had higher total fertility over 30 d as compared to INV/INV females, yet STD/STD females did not differ from either of these karyotypes (Fig. 3A; Table 1). However, repeated-measures MANOVA of the fertility trajectories over time revealed an effect of karyotype (i.e., a significant karyotype by time interaction; Table S1), with STD dominant over INV between days 21–30 (Fig. 3B). Consistent with this result, STD/STD females also had higher per-capita fecundity at 20–22 d than INV/INV females at 25 °C (Fig. 3C; Tables 1, S1), again with STD dominant over the recessive INV arrangement for increased reproductive output. At 18 °C, STD was partially dominant and hence INV had a partially recessive effect on fecundity (Fig. 3D; Table S1).

### INV reduces adult survival, yet male desiccation survival is overdominant

**Fig. 4:**
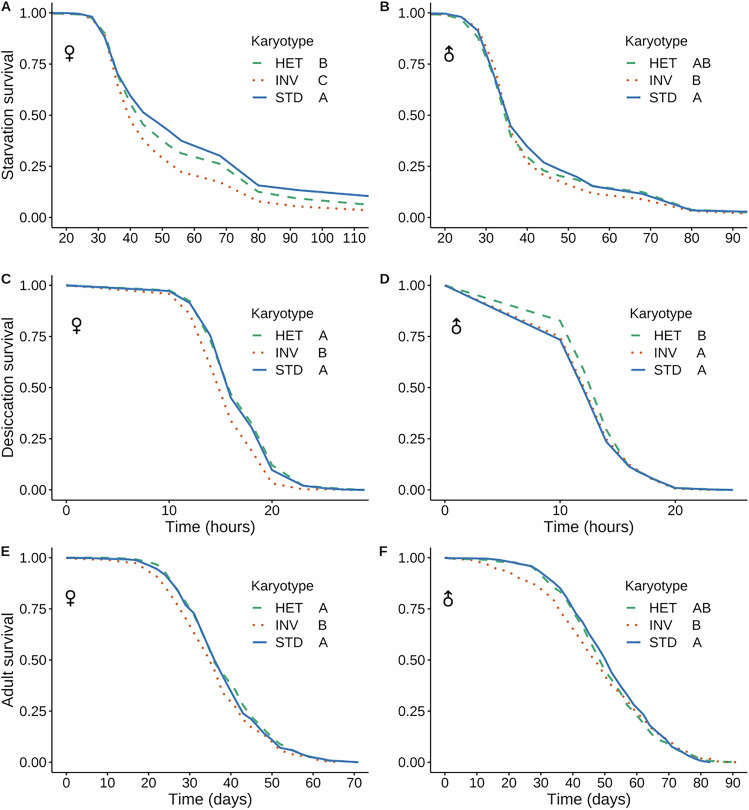
Effects of *3RP* on stress resistance and survival traits. Starvation resistance (fraction of flies alive upon starvation) for females (**A**) and males (**B**). Desiccation resistance (fraction of flies alive upon desiccation) for females (**C**) and males (**D**). Adult survival (lifespan; fraction of flies alive) for females (**E**) and males (**F**). Different letters for the karyotypes indicate significant pairwise differences (*P* < 0.05) after multiple-testing correction.

We also measured several traits related to stress resistance and survival (Fig. 4; Tables 1, S1). For starvation resistance (Fig. 4A, B), the STD/STD homokaryotype survived better than INV/INV (with additive effects in females but with the degree of dominance being indeterminable in males). In terms of desiccation resistance (Fig. 4B), we found that STD/STD females survived better than INV/INV females, with STD being dominant over INV. By contrast, in males (Fig. 4C), we observed overdominance of the heterokaryotype, indicating a sex-dependent change in the degree of dominance. Lastly, we measured adult lifespan and found that STD had a dominant effect over INV in both sexes, conferring longer lifespan (Fig. 4E, F; Tables 1, S1). Flies carrying one or two copies of the INV arrangement thus seem to be generally more stress-susceptible and shorter-lived than STD flies, with the notable exception of overdominance for male desiccation resistance.

### The *3RP* polymorphism exerts parental effects on fitness components

As we had used reciprocal crosses between the two homokaryotypes to generate both STD/INV and INV/SDT heterokaryotypes (see Materials and Methods), we were also able to ask whether there might be any parental effects (e.g., Mousseau and Fox 1998) associated with the inversion. Indeed, we observed several such parental (maternal and paternal) effects (see Supplementary Information, Figs. S3 and S4; also cf. Table S1). In terms of maternal effects, we observed that male (but not female) offspring produced by STD mothers had significantly larger wings as adults than offspring from INV mothers, independent of the paternal karyotype (Fig. S3A). Similarly, female (but not male) offspring of STD mothers showed greater survival upon desiccation as adults than female offspring of INV mothers, independent of the karyotype of the fathers (Fig. S3B). We also found several paternal effects. Female offspring of STD fathers had higher age-specific fecundity at 25 °C than daughters of INV fathers, independent of maternal karyotype (Fig. S4A). A similar pattern was seen for female dry weight, with daughters of STD fathers being heavier than those of INV fathers, independent of the karyotype of the mothers (Fig. S4B). Finally, daughters and sons of STD fathers survived desiccation as adults better than offspring of INV fathers, independent of whether the mothers were STD or INV (see Fig. S4C, D). Thus, despite clear patterns of dominance when considering the ‘average’ or ‘pooled’ heterokaryotype (see Table 1), we found substantial differences between the STD/INV and INV/SDT heterokaryotypes for several traits that are indicative of parental effects. As we discuss below, such parental effects can, at least in principle, contribute to the maintenance of polymorphism under conditions of temporally varying selection by leading to a temporal paternal ‘storage effect’ (Yamamichi and Hoso 2017; see below).

## Discussion

### Heterokaryotype superiority likely helps to maintain the polymorphism

A central finding of our study is that *3RP* exhibits marked heterokaryotype advantage (phenotypic overdominance) for several pre-adult fitness components (hatchability at 25 °C, pupal survival at 18 °C, viability, and development time) as well as for male desiccation resistance. These observations are notable as, with a small handful of exceptions (e.g., Watanabe and Watanabe 1973; Kamping and Van Delden 1999), practically nothing is known yet about heterokaryotype superiority for inversions in *D. melanogaster* (reviewed in Lemeunier and Aulard 1992; Kapun and Flatt 2019).

Our results for pre-adult fitness components, especially those for development time, suggest that the heterokaryotype might be a better larval competitor than the INV/INV and STD/STD homokaryotypes. In support of this interpretation, several studies in *Drosophila* have found that faster development often confers improved larval competitive ability (Chiang and Hodson 1950; Bakker 1962, 1968; De Jong 1976; Nunney 1983; de Miranda and Eggleston 1988; Mueller 1988; Grainger et al. 2021).

Interestingly, faster development and/or higher egg-to-adult survival of inversion heterokaryotypes has been observed in several *Drosophila* species before, including in *D. pseudoobscura* (Moos 1955), *D. persimilis* (Spiess and Schuellein 1956; Spiess 1958), and *D. pavani* (Brnic et al. 1969). Somewhat similar to our findings, Barnes (1983) observed heterokaryotype superiority for *3RP* for viability; yet, Barnes’ results were obtained in the context of a selection experiment for increased DDT resistance, which considerably complicates and confounds the interpretation of the ‘normal’ effects of the *3RP* inversion.

The cases of heterokaryotype advantage observed here likely make an important contribution to the maintenance of the *3RP* polymorphism. As some of these heterotic effects (i.e., for hatchability and pupal survival) depend on temperature, there might also be scope for a contribution of GxE interactions towards maintaining the polymorphism (Felsenstein 1976; Gillespie and Turelli 1989).

While several of the observed heterotic effects were small (~1–3% advantage over the homokaryotypes for hatchability and development rate), the effects on viability were quite substantial (~6–11%; see Table 2). It should be noted, however, that for some fitness components even seemingly small effects can be biologically quite important: for example, using numerical analyses of the Euler-Lotka equation, Lewontin (1965) found that a 10% increase in developmental rate can have approximately the same effect on fitness as a 100% increase in fertility, suggesting that a mutation that accelerates development by a specific fraction might confer a 10 times higher advantage than one that increases fertility by the same fraction (also see discussion in Green and Painter 1975).

### An ecological conjecture regarding pre-adult heterokaryotype advantage

Rapid development and higher larval competitive ability might be advantageous in variable environments. In their study of a community of *Drosophila* species in tropical Panama, Sevenster and van Alphen (1993b) found that some fly species exhibit a ‘fast’ life-history strategy (characterized by rapid development and short adult lifespan), whereas others have a ‘slow’ life history (with longer development and lifespan). Their results, also supported by mathematical modeling (Sevenster and van Alphen 1993a), indicate that ‘fast’ species have a competitive advantage when fallen, decaying fruit (i.e., the flies’ feeding, oviposition, and larval developmental sites) is seasonally abundant and when competition for access to feeding and oviposition substrate is strong. Given that in *Drosophila* generation time is short relative to the time scale of fluctuations in ephemeral resource (fruit) abundance, competition is exploitative, and faster development confers an advantage as it allows larvae to complete development before a food patch is exhausted (Sevenster and van Alphen 1993a, 1993b; also see Chiang and Hodson 1950; Bakker 1962, 1968; De Jong 1976; Nunney 1983; de Miranda and Eggleston 1988; Mueller 1988; Krijger et al. 2001; Frank (2022)). By contrast, while the ‘slow’ life-history strategy has reduced larval competitive ability, its higher adult survival might enable it to reach new breeding sites in space and time when such sites are rare and larval competition is weak (Sevenster and van Alphen 1993a, 1993b). Consistent with such a ‘slow’ strategy, the STD arrangement confers a longer lifespan as compared to the INV arrangement (also see Durmaz et al. 2018). Such ‘slow’ vs. ‘fast’ differences in life-history strategies can promote the coexistence of different species (or of different intraspecific genotypes) that exploit the same resources in variable environments (see Chesson 1985 and discussion in Sevenster and van Alphen 1993a, 1993b).

It is thus tempting to conjecture that the observed heterokaryotype superiority and such a ‘slow’ vs. ‘fast’ life-history dichotomy might have fostered the establishment of the *3RP* polymorphism and its maintenance under conditions of environmental variability. Consistent with this conjecture, *In(3R)P* originated in tropical sub-Saharan Africa (Corbett-Detig and Hartl 2012; Kapun et al. 2023), and the native ancestral range of *D. melanogaster* in southern-central Africa is known to be characterized by seasonally dry Miombo and Mopane woodlands (e.g., White 1983), with highly pronounced wet-dry season fluctuations in fruit abundance, temperature and precipitation (Mansourian et al. 2018; Sprengelmeyer et al. 2020; also cf. Walker 1995). These temporal fluctuations include the seasonally variable availability of Marula fruit (*Sclerocarya birrea*), to which *D. melanogaster* is very strongly attracted (Mansourian et al. 2018; P. Schmidt and T. Flatt, personal field observations in Zambia). Notably, the frequency of *3RP* has been found to fluctuate seasonally in several populations around the world, but whether this is due to fluctuations in resource abundance or other environmental factors is unclear (Inoue 1979; Stalker 1980; Masry 1981; Sanchez-Refusta et al. 1990; Kapun et al. 2016; Machado et al. 2021; Lange et al. 2022).

A caveat regarding the above conjecture about larval competitive ability is that we did not directly measure this trait, and our larval life-history assays were carried out at relatively low larval densities, i.e., relatively uncrowded conditions that presumably do not impose strong competition and density dependence.

Given the parallel clinal distribution of *3RP* on multiple continents outside of Africa (see Lemeunier and Aulard 1992; Kapun and Flatt 2019), the above scenario appears, at first glance, to be at odds with the fact that the inversion is absent (i.e., fixed for the STD arrangement) in temperate high-latitude areas characterized by pronounced seasonality, including major changes in resource (fruit) abundance and harsh winters. This absence of the INV arrangement in temperate areas could imply that it might be selected against under cool conditions (Kapun et al. 2023; also cf. Aulard et al. 2002; Pool et al. 2017). This idea is consistent with indirect evidence indicating that *3RP* might confer heat tolerance at the expense of cold tolerance (Anderson et al. 2003; also see Weeks et al. 2002). In direct support of this, we have previously confirmed that INV/INV homokaryotypes are more susceptible to cold shock-induced mortality than STD/STD flies (Durmaz et al. 2018).

More generally, the inversion polymorphism might be subject to life-history trade-offs and antagonistic pleiotropy (e.g., see Mérot et al. 2020; McAllester and Pool 2025, and references therein; also see discussion below). For example, recent work by McAllester and Pool (2025) has modeled a pleiotropic trade-off between male reproductive (display) success, a trait subject to negative frequency-dependent selection (NFDS), and survival (viability), and found that this mechanism can maintain inversion polymorphism.

Although we could not identify any fitness component for which the INV arrangement performs better than the STD arrangement, we note that the INV arrangement tends to confer a smaller body size (see Table 1; Fig. 2; also see Rako et al. 2006; Kapun et al. 2016b; Durmaz et al. 2018). This is interesting considering a recent study by Rao et al. (2025) who found that smaller-sized flies have a significant fitness advantage under conditions of adult crowding, with substantially reduced mortality and increased fecundity, hence suggesting that ‘bigger is not always better’.

### Potential mechanisms underlying overdominance

What might underpin the observed phenotypic patterns of heterozygote advantage? In principle, multiple genetic mechanisms can give rise to phenotypic overdominance (cf. Van Dooren 2000).

First, when the inversion arose, classical single-locus overdominance (OD) could have been generated de novo by mutational breakpoint effects or by the ancestral STD being fixed for one allele and the INV chromosome being fixed for an alternative allele at an overdominant locus (Kirkpatrick and Barton 2006; Kirkpatrick 2010; Durmaz et al. 2020). Such single-locus OD seems rather unlikely as an explanation, given the large number of fitness components affected by the *3RP* inversion, many of which are known to represent complex polygenic traits (see Flatt 2020) and would require an extreme degree of life-history pleiotropy (also see below).

Second, another possibility might be that the observed heterosis is due to pseudo-overdominance (POD) or associative overdominance (AOD) (Sturtevant, Mather (1938); Frydenberg 1963; Ohta and Kimura 1969; Ohta 1971). POD is the phenomenon whereby two haplotype blocks in a heterozygous state contain linked (partially or fully) recessive deleterious mutations in repulsion so that they are reciprocally masked, thus giving the appearance of single-locus OD. Under the related process of AOD, a polymorphic neutral locus appears to be subject to heterozygote advantage because of linkage disequilibrium (LD) with an OD locus or because of selection against recessive deleterious mutations. However, recent theory indicates that POD or AOD are unlikely to lead to superiority of inversion heterokaryotypes unless the INV and STD arrangements are about equal in frequency; also, these processes are expected to generate much smaller net fitness effects than those empirically observed, e.g., in *D. pseudoobscura* or *C. frigida* inversions (Charlesworth 2024; also cf. Wright and Dobzhansky 1946; Dobzhansky 1947; Anderson and Watanabe 1997; Mérot et al. 2020).

As Florida populations are subject to admixture from both African and European populations (Kao et al. (2015); Bergland et al. 2016; cf. discussion in Flatt 2016), and as the INV arrangement locally reaches frequencies of ~30–50% (Kapun et al. 2016a; Kapun and Flatt 2019), this situation could potentially create patterns of AOD/POD; for instance, INV heterokaryotypes could be protected against ancestry-related fitness-reducing epistatic interactions. The existence of such fitness-reducing epistatic interactions between African and European alleles has been reported from North American populations before (Lachance and True 2010; Kao et al. (2015); Kao et al. (2015); Pool 2015). While we cannot exclude the possibility that AOD/POD might contribute to the maintenance of the inversion polymorphism in the Florida populations examined here, or that it might in part confound the observed phenotypic effects reported here, it is improbable that AOD/POD can confer sufficient heterotic advantage to a newly arisen inversion for it to establish itself and spread to intermediate frequency in a large population (e.g., see discussion in Berdan et al. 2023; Charlesworth 2024). Thus, AOD/POD alone is unlikely to provide a sufficient, general explanation for the fact that *3RP* represents a balanced polymorphism in low-latitude populations around the world, including in ancestral-range populations in southern central Africa (see Kapun et al. 2023). If AOD/POD were to operate, its effects would be secondarily superimposed on the primary, main mechanism of balancing selection that has established the inversion polymorphism. Moreover, calculations in our previous work suggest that the ancestry (admixture) cline observed along the North American east coast (Bergland et al. 2016) cannot readily explain the steep inversion frequency cline between Florida and Maine (Kapun et al. 2016a). Ultimately, to disentangle the effects of ancestry from the karyotypic effects, it will be important to perform phenotypic assays on INV and STD karyotypes in other non-admixed (e.g., ancestral African) populations.

Third, an underlying additive genetic architecture at a single locus or across multiple linked loci could give rise to OD at a higher, more ‘integrated’ phenotypic level if the mapping between gene action and phenotype (or fitness) is nonlinear (see Van Dooren 2000; Omholt et al. 2000; cf. Crow 1952; see Hall and Wills 1987 for a concrete example). This possibility cannot be ruled out as a new inversion might capture a haplotype containing multiple beneficial loci with additive effects on fitness (Kirkpatrick and Barton 2006).

Fourth, the observed patterns of phenotypic OD could arise from multi-locus heterozygote advantage due to epistatic balancing selection acting on two or more linked OD loci in the heterokaryotype (i.e., the simplest version of Dobzhansky’s ‘coadaptation’ mechanism; Dobzhansky 1949, 1950, 1951, 1970; see Charlesworth 1974; also cf. Charlesworth and Charlesworth 1973; Schaeffer et al. 2003; Charlesworth and Flatt 2021; Kapun et al. 2023; Berdan et al. 2023).

To our mind, this is the most parsimonious scenario. First, multiple linked OD loci would explain the existence of multiple traits exhibiting phenotypic OD quite neatly. Such OD loci could also exhibit pleiotropic effects on fitness components. While we cannot rule out that all the effects of the inversion are due to a single, highly pleiotropic locus that exhibits OD (or marginal OD), this possibility strikes us as unlikely as the inversion affects a dozen or so complex, polygenic fitness components. Second, the simplest 2-locus model of epistatic ‘coadaptation’ with fixed selection coefficients (see Charlesworth 1974; Charlesworth and Flatt 2021) generates ‘apparent’ NFDS, with multiple frequency equilibria or quasi-equilibria whose attainment depends on history, the initial conditions of the population, and/or the local environment: this could explain why different low-latitude populations of *3RP* exhibit different intermediate frequencies (see Kapun and Flatt 2019; Kapun et al. 2023) and would also be consistent with a previous experiment reporting evidence for NFDS acting on *3RP* (Nassar et al. 1973; M. Paris, T. Rey, and T. Flatt, unpublished experimental data). Notably, the fact that *In(3R)P* is often found at markedly different intermediate frequencies in different populations (global average frequency ~15%; frequency in ancestral range populations ~10–12%; frequencies above ~60% in some other locations; e.g., see Kapun and Flatt 2019; Kapun et al. 2023) is clearly at odds with a single-locus OD mechanism which would generate only a single equilibrium frequency.

Ultimately, dissecting the genetic architecture of the fitness effects of the *3RP* inversion will require fine-scale mapping, e.g., using CRISPR/Cas9-based approaches (e.g., see discussion in Berdan et al. 2023).

### The phenotypic effects of the homokaryotypes are consistent across studies

How do our results, based on a panmictic population cage approach, compare to prior work? In two previous studies, we used isochromosomal lines to compare the homozygous effects of homokaryotypic STD vs. INV lines without investigating heterokaryotypes and without using the population cage approach (Kapun et al. 2016b; Durmaz et al. 2018). In terms of differences between homokaryotypes, our results for wing area, femur length, desiccation survival, and lifespan are entirely consistent with our previous findings, with the same directionality of the effects. We also note that Rako et al. (2006), using *3RP* INV vs. STD flies isolated from the Australian cline, found qualitatively identical effects to ours for wing area. Moreover, our previous failure to find differences between the homokaryotypes for development time and viability in Kapun et al. (2016b) is completely consistent with our finding here that the two homokaryotypes do not differ for these traits, with both traits exhibiting heterokaryotype superiority. This suggests that the phenotypic effects of *3RP* are likely robust, repeatable, and independent of the details of the experimental design; it might also indicate that the underlying phenotypic differences between the arrangements are genetically fixed.

### The inversion polymorphism might also be subject to antagonistic selection

In addition to several cases of overdominance, we also observed some instances of changes in the degree of dominance (see Table 1) that are indicative of context-dependent antagonistic selection involving beneficial dominance reversals. Under this form of selection, which can manifest itself as antagonistic pleiotropy, sexually antagonistic selection, or as spatially or temporally varying selection, a given allele is partially or fully dominant in the specific context (e.g., sex, environment, niche, season, life history trait) in which it is selectively favored but partially or fully recessive in the alternative context (e.g., in the other sex, the alternative environment) in which it is deleterious (reviewed in Connallon and Chenoweth 2019; Grieshop et al. 2024). Such a situation can lead to ‘marginal’ overdominance, i.e., an average net advantage of the heterozygote across the different contexts and thus contribute to maintaining variation (cf. Grieshop et al. 2024).

Consistent with such a mode of selection, we found that the INV arrangement was dominant for small wing size but recessive for femur length in females, perhaps exemplifying antagonistic pleiotropy with dominance reversals (Rose 1982, 1985; Curtsinger et al. 1994; Charlesworth and Hughes 2000). We note, however, that the concept of antagonistic pleiotropy only applies, in a strict sense, to a single-locus situation; it is only applicable if the whole inversion is being viewed as a single locus with two Mendelian alternative alleles (STD vs. INV) or if a single pleiotropic locus within the inversion is involved. Similarly, between the sexes, we observed a dominance reversal for femur length, with the INV arrangement being dominant in females but recessive in males.

In a similar vein, theoretical work has shown that temporally fluctuating selection involving a regime of seasonal reversals of dominance can maintain polymorphism (Wittmann et al. 2017; see Karageorgi et al. 2025 for an empirical example; also cf. Brud 2025) – this possibility is particularly interesting since *3RP* frequency is often subject to local seasonal fluctuations (e.g., Inoue 1979; Masry 1981; Sanchez-Refusta et al. 1990; Kapun et al. 2016; Machado et al. 2021; Lange et al. 2022), as already mentioned further above.

We also identified other changes in the degree of dominance between different traits, the sexes, and temperature regimes (Table 1); however, these changes were, strictly speaking, not actual *reversals* of dominance. For instance, the INV arrangement was recessive for desiccation survival in females but overdominant in males, and age-specific daily fecundity changed from partially recessive (*h* = 0.4) at 18 °C to fully recessive (*h* = 0) at 25 °C (Table 1). Interestingly, a theoretical study by Brud (2025) suggests that also nonreversing, context-dependent changes in dominance, e.g., between seasons, can stabilize polymorphism.

Together, our results indicate that antagonistic selection might potentially contribute to maintaining the *3RP* polymorphism. This would be consistent with the results of Mérot et al. (2020), who observed that ‘antagonistically pleiotropic’ effects with beneficial dominance reversals contribute to maintaining an inversion polymorphism in seaweed flies, even though the genetic architecture underlying this pleiotropic effect (i.e., whether is based on single pleiotropic locus) remains unclear (see discussion above; also see McAllester and Pool 2025).

### The *3RP* polymorphism is associated with parental effects

Interestingly, when comparing heterokaryotypic flies from reciprocal female-to-male vs. male-to-female crosses (i.e., STD/INV vs. INV/STD) to the two homokaryotypes, we observed clear parental effects (e.g., Mousseau and Fox 1998; Qvarnström and Price 2001). Male wing area and female desiccation resistance were subject to maternal effects, whereas female dry weight, age-specific fecundity at 25 °C, and both female and male starvation resistance were affected by paternal effects. Notably, Barnes (1984) also found evidence for a maternal effect associated with *3RP*, namely for viability, unlike what we have observed here; however, the findings of Barnes are difficult to interpret, as they were obtained in the context of a laboratory selection experiment for DDT resistance.

The parental effects that we have observed could well be related to sexual selection (e.g., Qvarnström and Price 2001) or sexual antagonism (e.g., García-Roa et al. 2024); importantly, they could play a role in the maintenance of the inversion polymorphism: theoretical work by Yamamichi and Hoso (2017) has demonstrated that parental effects can facilitate the maintenance of genetic polymorphism under conditions of temporally varying selection. This is because parental effects cause a mismatch between genotype and phenotype; in turn, this mismatch can buffer the effects of selection on allele frequencies, resulting in a temporal parental ‘storage’ effect that can maintain variation (Yamamichi and Hoso 2017; also cf. Dey et al. 2016). Again, it is noteworthy in this context that the frequency of *3RP* is subject to seasonal fluctuations that are consistent with temporally varying selection (see references above).

## Conclusions

Our results indicate that the well-known, cosmopolitan *In(3R)Payne* inversion polymorphism of *D. melanogaster* might be subject to several forms of balancing selection, including overdominant selection, antagonistic selection involving context-dependent dominance reversals between different traits and the sexes, genotype-by-environment interactions, and parental effects. The action of multiple modes of phenotypic balancing selection could explain why previous studies have found that this inversion polymorphism is likely affected by several selective forces, including, for instance, spatially varying (clinal) selection, temporally (seasonally) varying selection, and negative frequency-dependent selection (e.g., see discussion in Kapun and Flatt 2019; Kapun et al. 2023). While it is clear from a theoretical perspective that these forms of balancing selection are not mutually exclusive and can interact with each other (e.g., Faria et al. 2019; Chevin et al. 2022; Westram et al. 2022; Berdan et al. 2023), little is understood about their actual interplay.

## Supplementary information

Supporting Information file

Table S1

Table S2

## Data Availability

Code is provided in Table S1, and raw data are given in Table S2. The raw data are also publicly available at Dryad: 10.5061/dryad.bvq83bkn7
