# Supplementary material for: Multiple forms of balancing selection maintain inversion polymorphism
Source: Heredity (Edinb). 2025 Jul 17;135(3):138–51. doi: 10.1038/s41437-025-00780-y (PMC13031870; doi:10.1038/s41437-025-00780-y)
Supplement: Supplementary file 1 — Supporting Information file [file 41437_2025_780_MOESM1_ESM.docx]

**Supporting Information**

for the article entitled:

‘Multiple Forms of Balancing Selection Maintain Inversion Polymorphism’

Authors:

Margot Paris, Esra Durmaz Mitchell, Envel Kerdaffrec, Doriane Rubin, Cécile Spichtig, Felicitas Zurbriggen, Joël Becker, Hannah Augustijnen, Harshavardhan Thyagarajan, Eliane Zinn, Fanny Gagliardi, Elliot Gobet, Tristan Rey, Yvan Rime, Sofia Ribeiro Machado, Jeremias Bachmann, Noemi Sgammeglia, Paul Schmidt, and Thomas Flatt

Correspondence: [thomas.flatt@unifr.ch](mailto:thomas.flatt@unifr.ch)

**Supporting Figures - Overview**

The figures below represent supplementary results mentioned in the main text of our paper. For further details, please see the main text and the Materials and Methods section.

- **Fig. S1:** Details of morphometric measurements Page 2
- **Fig. S2:** Effects of *In(3R)Payne* on pupal survival Page 3
- **Fig. S3:** Maternal effects of *In(3R)Payne* Page 4
- **Fig. S4:** Paternal effects of *In(3R)Payne* Page 5

**
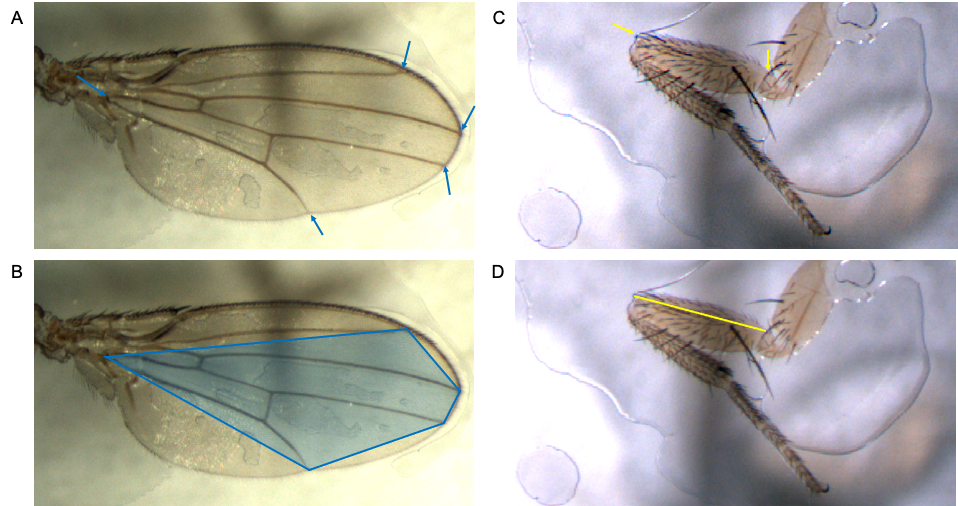
**

**Fig. S1. Details of morphometric measurements.** The figure illustrates how morphometric measurements were acquired. **(A)** Positions of the 5 landmarks used for wing area measurements (see arrows). **(B)** The polygon between the 5 landmarks was used as a proxy for the wing area. **(C)** Position of the 2 landmarks used for measurements of femur length. **(D)** Femur length is defined as the yellow line between the 2 landmarks. See the Materials and Methods section for further details.


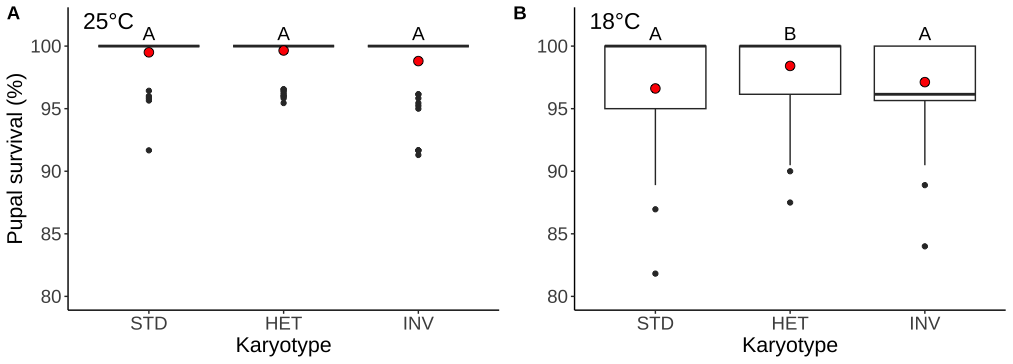


**Fig. S2. Effects of *In(3R)Payne* on pupal survival**. Pupal survival (=pupa-to-adult survival) at 25°C **(A)** and 18°C **(B)**. For each karyotype, mean values are represented as red filled circles. Different letters denote significant pairwise differences (*P* < 0.05) between karyotypes after multiple-testing correction. See the article’s main text for further details; also see Table S1.


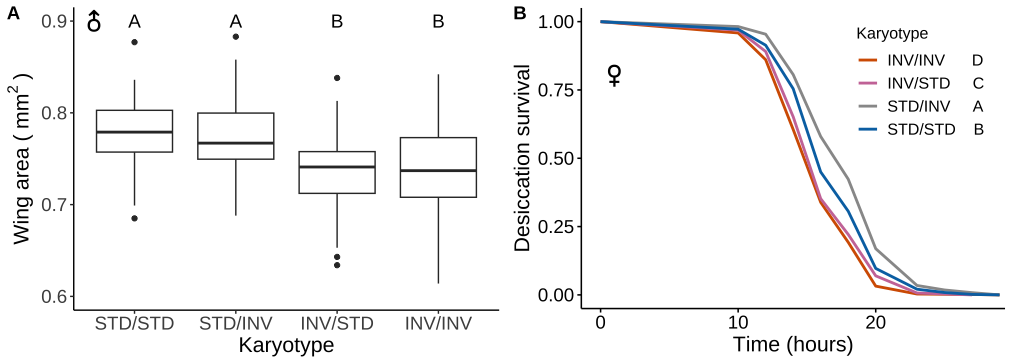


**Fig. S3. Maternal effects of *In(3R)Payne***. Maternal effect on male wing area **(A)** and female desiccation resistance **(B)**. Different letters denote significant pairwise differences (*P* < 0.05) between karyotypes after multiple-testing correction. In the reciprocal crosses, STD/INV designates a STD dam [mother] crossed to an INV sire [father], and INV/STD designates an INV dam [mother] crossed to a STD sire [father]. See the article's main text for further details; also see Table S1.


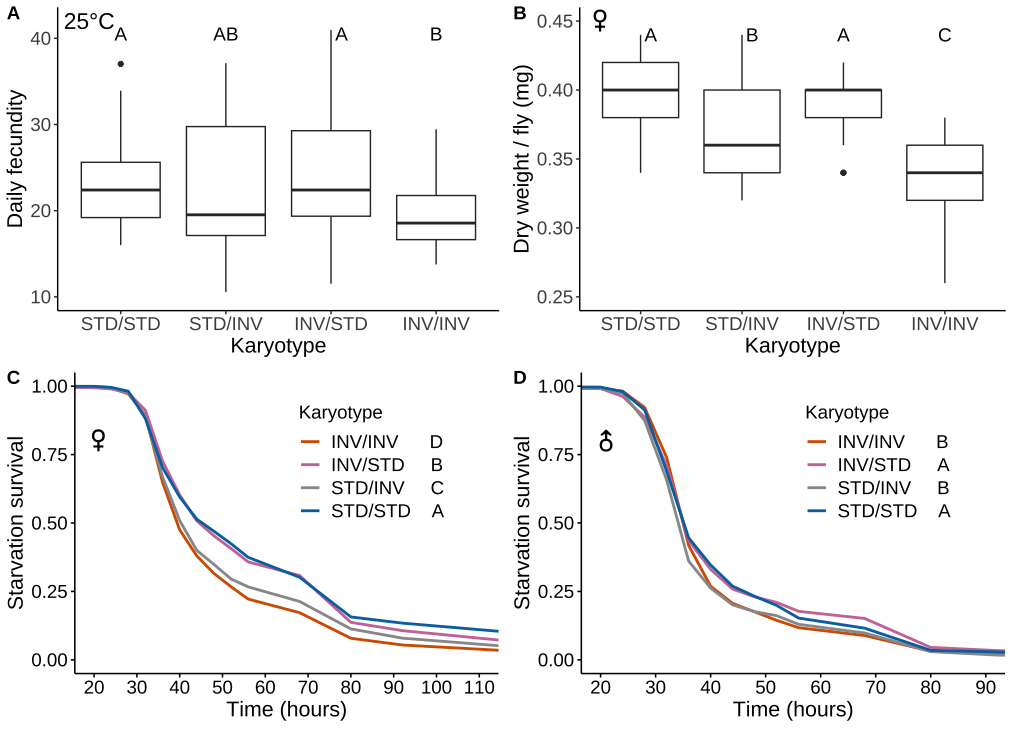


**Fig. S4. Paternal effects of *In(3R)Payne***. Paternal effects on age-specific daily fecundity at 25°C **(A)**, female dry weight **(B)**, starvation resistance in females **(C)** and males **(D)**. Different letters denote significant pairwise differences (*P* < 0.05) between karyotypes after multiple-testing correction. In the reciprocal crosses, STD/INV designates a STD dam [mother] crossed to an INV sire [father], and INV/STD designates an INV dam [mother] crossed to a STD sire [father]. See the article's main text for further details; also see Table S1.
